# The Effect of Oral Isotretinoin on Quality of Life in Patients With Acne Vulgaris: A Systematic Review and Meta-Analysis of Patient-Reported Outcomes

**DOI:** 10.7759/cureus.116205

**Published:** 2026-09-14

**Authors:** Abdulelah A Albaqami, Sultan A Albqami

**Affiliations:** 1 Department of Dermatology, King Abdulaziz Hospital, Bisha, SAU; 2 Department of Preventive Medicine, Jazan Health Cluster, Jazan, SAU

**Keywords:** acne vulgaris, dermatology life quality index, isotretinoin, meta-analysis, quality of life, systematic review

## Abstract

Acne vulgaris is a highly prevalent chronic inflammatory skin disorder that can adversely affect psychological well-being, social functioning, and overall quality of life. Oral isotretinoin is widely regarded as an effective treatment for moderate to severe acne, yet its influence on patient-reported quality-of-life outcomes remains incompletely characterized because published studies have reported variable findings. We performed a systematic review and meta-analysis following PRISMA (Preferred Reporting Items for Systematic Reviews and Meta-Analyses) 2020 recommendations. PubMed, Scopus, Web of Science, the Cochrane Library, and Google Scholar were searched from database inception to April 5, 2026. Eligible studies included patients with acne vulgaris treated with oral isotretinoin and reporting quality-of-life outcomes using validated instruments. Risk of bias was evaluated using ROBINS-I (Risk Of Bias In Non-randomized Studies of Interventions) for non-randomized studies and the Cochrane Risk of Bias 2 (RoB 2) for the randomized trial. For studies reporting sufficient independent Dermatology Life Quality Index (DLQI) data, pooled effect estimates were calculated using a random-effects model.

Fourteen studies with varying designs, settings, and patient populations met the inclusion criteria for the systematic review. Across studies, oral isotretinoin treatment was generally associated with improved quality of life, with the most consistent benefits observed with dermatology-specific and acne-specific instruments. Four studies contributed sufficient independent data to the quantitative synthesis. Meta-analysis showed a significant reduction in DLQI scores following isotretinoin treatment, corresponding to improved quality of life (standardized mean difference (SMD) = −1.49, 95% confidence interval (CI): −2.48 to −0.51). Heterogeneity was substantial (I² = 98.1%). In exploratory subgroup analyses, improvements were observed in both mixed-severity and severe or treatment-resistant acne populations, with no statistically significant difference between subgroups.

Oral isotretinoin appears to be associated with meaningful improvements in quality of life among patients with acne vulgaris, particularly when assessed using dermatology- or acne-specific instruments. However, the substantial heterogeneity and predominance of non-randomized studies warrant cautious interpretation of the pooled effect. Further well-designed prospective and randomized studies using standardized quality-of-life measures and longer follow-up are needed to better establish the magnitude and durability of these benefits.

## Introduction and background

The skin is a complex organ composed of the epidermis, dermis, and subcutaneous tissue, functioning as a protective barrier and playing a key role in thermoregulation and immune defense. Among dermatological disorders, acne vulgaris is one of the most prevalent chronic inflammatory diseases of the pilosebaceous unit [1]. It commonly presents with comedones, papules, pustules, nodules, and, in severe cases, cystic lesions, primarily affecting the face, chest, back, and shoulders. Globally, acne affects approximately 9.4% of the population, ranking as the eighth most common disease worldwide and the third most frequent skin condition overall [2]. Epidemiological data indicate that nearly 85% of adolescents experience acne, although it also affects children and adults [3].

Although acne is not life-threatening, its psychological and social burden is substantial [4]. Multiple studies have demonstrated associations between acne and reduced self-esteem, social withdrawal, anxiety, and depressive symptoms, leading to measurable impairments in quality of life [5-7]. Quality-of-life instruments such as the Dermatology Life Quality Index (DLQI) and Cardiff Acne Disability Index (CADI) have consistently shown a moderate to severe impact on quality of life in untreated or poorly controlled acne [8,9]. The burden is particularly pronounced in moderate to severe disease and in adolescents and young adults, who represent a population already vulnerable to psychosocial stressors [10].

Treatment strategies for acne include topical therapies, systemic antibiotics, and oral isotretinoin. Oral isotretinoin remains the most effective treatment for severe and recalcitrant acne, with remission rates exceeding 80% in many clinical cohorts [11]. It is the only therapy that targets all major pathogenic mechanisms of acne, including sebaceous gland activity, follicular keratinization, inflammation, and Cutibacterium acnes colonization [12]. Standard dosing regimens range from 0.5 to 2 mg per kg per day over four to six months, often achieving long-term disease control [13].

Despite its efficacy, isotretinoin is associated with a wide range of adverse effects. Common effects include mucocutaneous dryness, musculoskeletal symptoms, and biochemical abnormalities, while more serious concerns include teratogenicity and potential psychiatric effects [2]. Between 1982 and 2000, the United States Food and Drug Administration received over 400 reports of depression, suicidal ideation, or suicide in patients receiving isotretinoin, placing it among the drugs most frequently associated with psychiatric safety reports [14]. Although causality remains debated, these concerns have raised questions regarding the overall impact of isotretinoin on patients’ quality of life.

Importantly, isotretinoin may improve quality of life through reductions in acne severity while also having the potential to adversely affect quality of life through treatment-related adverse effects, including psychological symptoms [15]. Existing studies have reported variable findings, with some demonstrating substantial improvements in quality of life and others reporting more limited or domain-specific changes. These differences may be influenced by study design, acne severity, treatment duration, isotretinoin dosing, and population characteristics. A comprehensive synthesis of the available evidence is therefore needed to better characterize the overall association between oral isotretinoin treatment and quality-of-life outcomes in patients with acne.

## Review

Methods

Study Design

This study was conducted as a systematic review and meta-analysis in accordance with the PRISMA (Preferred Reporting Items for Systematic Reviews and Meta-Analyses) 2020 guidelines [16]. The objective of this review was to synthesize and quantitatively evaluate the evidence regarding the effect of oral isotretinoin therapy on quality-of-life outcomes in patients with acne. The review protocol was not registered in PROSPERO.

The research question was formulated using the PICO framework as follows: Population (P): Adolescents and adults diagnosed with acne vulgaris. Intervention (I): Oral isotretinoin therapy at any dose or for any treatment duration. Comparator (C): Baseline (pre-treatment) assessments and/or alternative acne treatments, including topical therapies or systemic antibiotics, where applicable. Outcome (O): Changes in quality of life measured using validated instruments, such as the DLQI [17], CADI [18], Skindex, the 36-Item Short Form Survey (SF-36) [19], the World Health Organization Quality of Life-BREF (WHOQOL-BREF) [20], or acne-specific quality-of-life scales [21].

Search Strategy

A comprehensive literature search was conducted across five electronic databases, including PubMed (MEDLINE), Scopus, Web of Science Core Collection, the Cochrane Library, and Google Scholar, from database inception to April 5, 2026. The search strategy combined controlled vocabulary terms and free-text keywords related to acne, isotretinoin, and quality-of-life outcomes. Key concepts included terms related to acne (e.g., acne, acne vulgaris), oral isotretinoin (e.g., isotretinoin, oral isotretinoin, 13-cis-retinoic acid, Accutane, Roaccutane), and quality-of-life outcomes (e.g., quality of life, DLQI, CADI, Skindex, Acne-QoL, SF-36, WHOQOL-BREF). Boolean operators (AND/OR) were used to optimize search sensitivity. Searches were limited to studies published in English. The complete database-specific search strategies are provided in Appendix 1. Reference lists of the included studies were also manually screened to identify additional potentially eligible studies.

Eligibility Criteria

Studies were included if they enrolled patients diagnosed with acne vulgaris and evaluated oral isotretinoin as a systemic treatment. Eligible studies were required to report quality-of-life outcomes assessed using validated instruments, including DLQI, CADI, Skindex, SF-36, WHOQOL-BREF, or acne-specific quality-of-life measures. Both randomized controlled trials and non-randomized observational designs were eligible, including prospective and retrospective cohort studies as well as before-and-after studies. To be included, studies had to provide sufficient quantitative data to describe changes in quality of life or permit extraction of relevant outcome measures.

Studies were excluded if they focused on non-acne populations or acneiform disorders, evaluated non-oral isotretinoin formulations or topical retinoids only, or did not report quality-of-life outcomes. Reviews, editorials, guidelines, and narrative commentaries were excluded unless they contained original, extractable patient-level or group-level data relevant to the outcomes of interest. Case reports, conference abstracts without full-text publication, animal or preclinical studies, and purely laboratory-based investigations were excluded. Studies that were unavailable in full text or published in languages other than English were also excluded.

Study Selection and Screening

All references retrieved were imported into Rayyan (https://www.rayyan.ai/) for duplicate removal and screening [22]. Duplicate records were identified and removed prior to screening. Two reviewers independently screened the titles and abstracts for eligibility based on predefined inclusion and exclusion criteria. The full texts of potentially eligible studies were then assessed independently by the same reviewers. Any disagreements were resolved through discussion and consensus.

Risk-of-Bias Assessment

Risk of bias was assessed using study design-specific, validated tools to ensure an appropriate and methodologically rigorous evaluation of internal validity. All non-randomized studies of interventions were assessed using the ROBINS-I (Risk Of Bias In Non-randomized Studies of Interventions) tool [23]. ROBINS-I is specifically designed to evaluate the risk of bias in observational studies that assess the effects of interventions and allows comparison of such studies to a hypothetical target randomized trial. The tool evaluates bias across seven predefined domains: bias due to confounding, bias in selection of participants into the study, bias in classification of interventions, bias due to deviations from intended interventions, bias due to missing data, bias in measurement of outcomes, and bias in selection of the reported result. Each domain was judged as low, moderate, serious, or critical risk of bias, and an overall risk-of-bias judgment was assigned based on the highest risk identified across domains.

The single randomized controlled trial included in the review was assessed separately using the Cochrane Risk of Bias 2 (RoB 2) tool [24], which was specifically developed for randomized trials. RoB 2 evaluates potential bias arising from the randomization process, deviations from intended interventions, missing outcome data, outcome measurement, and selective reporting. All risk-of-bias assessments were conducted independently by two reviewers. Any discrepancies were resolved through discussion and consensus to ensure consistency and accuracy.

Data Extraction and Synthesis

Data were extracted using a standardized data extraction form. Extracted variables included author and year of publication, country, study design, sample size, participant characteristics, acne severity, isotretinoin dosing regimen, comparator (if applicable), quality-of-life instruments used, assessment time points, and reported outcomes. Data extraction was conducted independently by two reviewers to ensure accuracy and completeness, with discrepancies resolved through discussion. When necessary, supplementary materials were consulted for clarification. A narrative synthesis was conducted for all included studies. Studies reporting DLQI outcomes with sufficient quantitative data were eligible for inclusion in the meta-analysis.

Statistical Analysis

Statistical analyses were performed using R software (version 4.3.3). For the quantitative synthesis, standardized mean differences (SMDs) with 95% confidence intervals (CIs) were calculated for changes in DLQI scores using a random-effects model to account for between-study variability. Statistical heterogeneity was assessed using Cochran’s Q test and quantified with the I² statistic. Assessment of publication bias was not performed, as the number of studies included in the quantitative synthesis (n = 4) was below the minimum recommended threshold of 10 studies [25]. Funnel plot asymmetry tests, including Egger’s regression and Begg’s rank correlation, are known to yield unreliable results when applied to small numbers of studies due to limited statistical power and an increased risk of misleading conclusions [25,26]. Therefore, consistent with methodological guidance, no formal evaluation of publication bias was undertaken.

Results

Study Selection

The literature search identified 835 records across five electronic databases: PubMed (n = 86), Scopus (n = 436), Web of Science (n = 238), the Cochrane Library (n = 25), and Google Scholar (n = 50). After removal of 226 duplicate records, 609 records were screened by title and abstract. Of these, 591 records were excluded because of wrong population (not acne vulgaris) (n = 250), wrong outcome (quality of life not reported) (n = 200), wrong intervention (not oral isotretinoin) (n = 86), or inappropriate publication type (review, editorial, commentary, or guideline) (n = 55). Eighteen reports were sought for retrieval, and all 18 were retrieved and assessed for eligibility. Four reports were excluded at the full-text stage because of insufficient data for extraction (n = 1), quality of life not reported quantitatively (n = 1), or wrong intervention (isotretinoin not oral or mixed treatment without separable data) (n = 2). Ultimately, 14 studies met the inclusion criteria and were included in the systematic review. The study selection process is illustrated in Figure 1.

**Figure 1:**
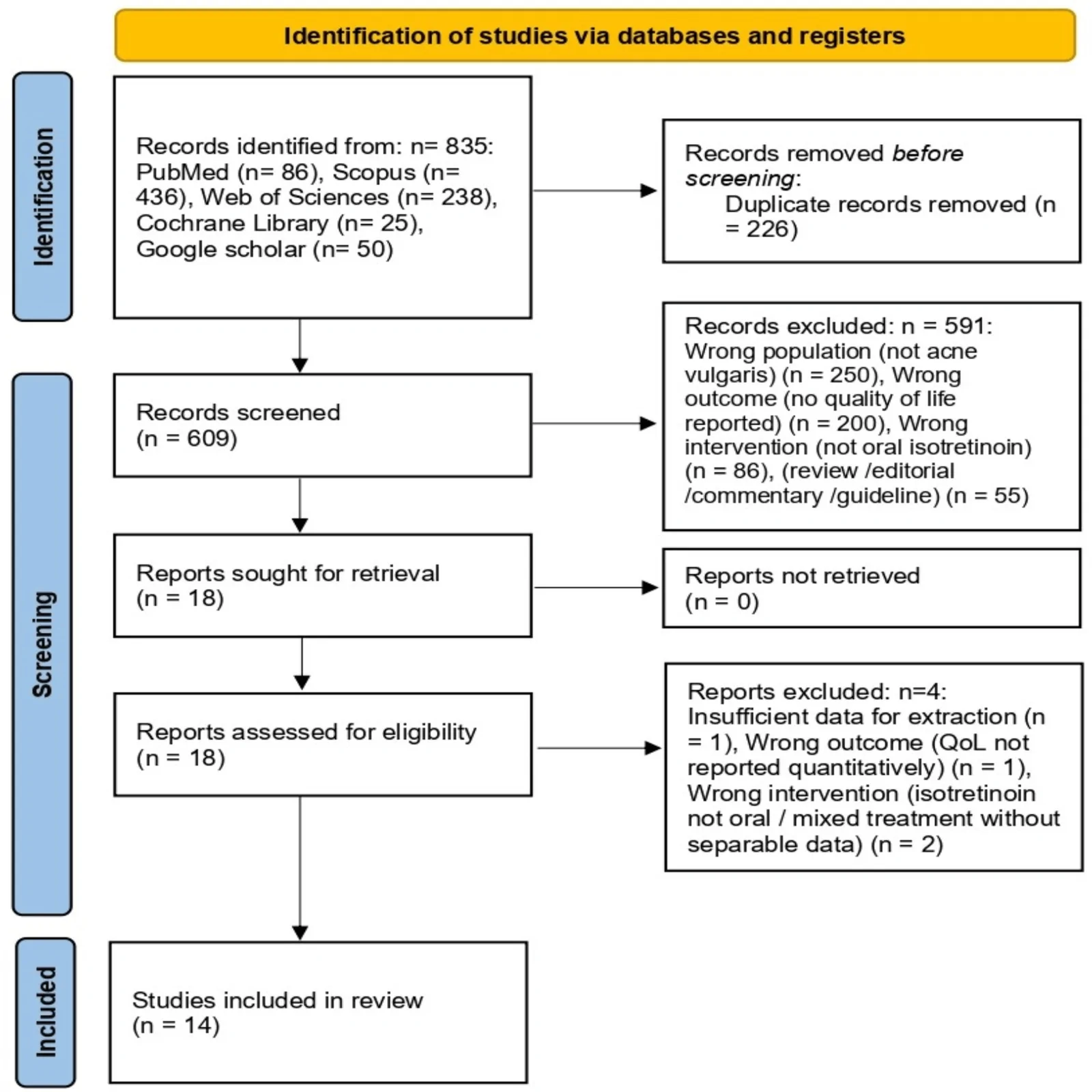
PRISMA 2020 flow diagram depicting study selection for the systematic review PRISMA: Preferred Reporting Items for Systematic Reviews and Meta-Analyses

Study Characteristics

The characteristics of the included studies are summarized in Table 1. The 14 studies were published between 2002 and 2024 and were conducted across Australia, Europe, Asia, the Middle East, and the United States. Study designs included one randomized controlled feasibility trial, six prospective controlled or cohort studies, four prospective before-after observational studies, and three retrospective cohort studies. Sample sizes of isotretinoin-treated participants ranged from seven to 346, with most studies enrolling young adults with a mean age between 20 and 23 years. The majority of participants had moderate to severe acne, although several studies focused specifically on severe or treatment-resistant acne. Oral isotretinoin dosing regimens varied across studies, most commonly ranging from 0.5 to 1.0 mg/kg/day, with cumulative doses generally ranging from approximately 100 to 150 mg/kg, although some studies used higher cumulative exposure. Comparator groups, when present, included topical therapies, oral antibiotics, or combined antibiotic-topical regimens.

**Table 1:** Characteristics of studies assessing the effect of oral isotretinoin on quality of life in patients with acne Note: Sample size refers to the isotretinoin-treated group unless otherwise stated. QoL assessment time points are reported as described in the individual studies. Where an exact follow-up time was not specified in the available data, this is indicated as “follow-up” or “pre- vs post-treatment.” Studies included randomized, prospective, and retrospective observational designs AGS: Acne Grading Scale; APSEA: Acne Patient Symptom Assessment; CADI: Cardiff Acne Disability Index; DLQI: Dermatology Life Quality Index; GAGS: Global Acne Grading System; QoL: quality of life; RAGS: Leeds Revised Acne Grading System; SF-36: Short Form-36 Health Survey; WHOQOL-BREF: World Health Organization Quality of Life–Brief Version

Study	Country	Study design	Sample size (n)	Participant characteristics	Isotretinoin regimen	Comparator	QoL instrument(s)	QoL assessment time points
Ng et al., 2002 [14]	Australia	Prospective non-randomized controlled study	174	Mean age 20.2 ± 5.8 years; 58.6% male; moderate–severe acne (93% AGS ≥ 4)	Oral isotretinoin starting at 40 mg/day, increased to 1.0 mg/kg/day; cumulative dose 120 mg/kg over 5–6 months	Antibiotic + topical therapy (n=41)	WHOQOL-BREF	Baseline; 1, 3, and 6 months
Hahm et al., 2009 [27]	South Korea	Prospective cohort study	38	Mean age 23 years; 11 males, 27 females; mild–severe acne (Leeds RAGS grades 1–7)	Oral isotretinoin 0.5–1.0 mg/kg/day for 8 weeks	None	APSEA (acne-related QoL)	Baseline; 2 and 8 weeks
Kaymak et al., 2009 [28]	Turkey	Prospective controlled (non-randomized) trial	36 (isotretinoin arm)	Mean age 20.61 ± 1.87 years; 11 males, 25 females; mild–severe acne	Oral isotretinoin 0.5–0.8 mg/kg/day for ≥ 20 weeks (cumulative ~100 mg/kg)	Topical therapy	DLQI	Baseline; 2 and 4 months
McGrath et al., 2010 [29]	United Kingdom	Prospective controlled study	65	Mean age ~20 years; acne patients attending dermatology clinics	Oral isotretinoin 0.5 mg/kg/day, increased to 1 mg/kg/day; cumulative dose ~120 mg/kg	Antibiotics + topical therapy	WHOQOL-BREF	Baseline; follow-up
Berg and Lindberg, 2011 [30]	Sweden	Retrospective observational cohort (structured outpatient clinic; semi-quantitative grading)	211	Mean age 21.6 years; 143 females, 68 males; mild–severe acne	Oral isotretinoin; dose not specified	Local therapy; oral antibiotics	DLQI	Baseline; 6 months
Cyrulnik et al., 2012 [31]	USA	Retrospective cohort with telephone QoL survey	80 (QoL data from 59)	Mean age 22.9 years (range 12–47); 68.8% females; severe nodulocystic acne	High-dose oral isotretinoin ≥ 1.3 mg/kg/day; mean 1.6 mg/kg/day; cumulative dose ~290 mg/kg; ~5–6 months	None	Acne-QoL	Pre- vs post-treatment telephone survey
Ergun et al., 2012 [32]	Turkey	Prospective multicenter observational study	63 enrolled; 48 completed	Mean age: females 22.6 ± 4.3 years, males 19.3 ± 3.9 years; severe, resistant acne	Oral isotretinoin 0.5 mg/kg/day initially, increased to 1 mg/kg/day; cumulative dose 130–150 mg/kg	None	CADI	Baseline; 1 month; 3 months; end of treatment
Yesilova et al., 2012 [33]	Turkey	Prospective cohort study	30	Mean age 22.6 years; acne vulgaris	Oral isotretinoin 0.5–1.0 mg/kg/day for 6 months	None	SF-36	Baseline; 6 months
Karadag et al., 2013 [34]	Turkey	Prospective before–after observational study	38	Mean age: females 21.3 years, males 19.4 years; severe acne vulgaris; no psychiatric history	Oral isotretinoin 0.5–1.0 mg/kg/day for ~24 weeks	None	SF-36	Baseline; 6 months
Marron et al., 2013 [35]	Spain	Prospective observational longitudinal study	346	Mean age 20.7 ± 2.1 years; 58.6% female; moderate acne; patients aged ≥ 16 years	Oral isotretinoin, weight-dependent dosing; cumulative dose 120 mg/kg over 30 weeks	None	DLQI; SF-36	Baseline; 30 weeks (end of treatment)
Fakour et al., 2014 [36]	Iran	Prospective before–after observational study	98	Mean age 22.0 ± 4.4 years; 38 males, 60 females; severe acne (GAGS)	Oral isotretinoin 0.5 mg/kg/day for 16 weeks	None	DLQI	Baseline; 16 weeks
Rea et al., 2018 [37]	Australia	Triple-blind randomized controlled feasibility trial	7 (isotretinoin arm)	Acne vulgaris patients eligible for systemic therapy; psychiatric history excluded	Oral isotretinoin 20 mg twice daily for 2 weeks	Doxycycline 50 mg twice daily	DLQI; WHOQOL-BREF	Baseline; 2 weeks
Secrest et al., 2020 [38]	USA	Longitudinal retrospective cohort study	57	Median age 17.2 years; moderate–severe acne	Oral isotretinoin titrated to cumulative dose ~120 mg/kg	None	Skindex-16	Monthly, from baseline to 6 months
Bhushan et al., 2024 [39]	India	Prospective observational follow-up study	85	Mean age 21.41 ± 4.39 years; 70.6% females; moderate to very severe acne (GAGS)	Oral isotretinoin 0.5 mg/kg/day for 6 months	None	DLQI; CADI	Baseline; 3 months; 6 months

Quality-of-Life Outcomes

Quality-of-life outcomes are detailed in Table 2. Across the fourteen included studies, a variety of validated QoL instruments were used, including the Dermatology Life Quality Index (DLQI), Cardiff Acne Disability Index (CADI), WHOQOL-BREF, SF-36, Skindex-16, Acne-QoL, and APSEA. Assessment time points ranged from two weeks to six months, with most studies evaluating outcomes at baseline and at least one post-treatment follow-up. Overall, studies generally reported improvements in quality of life following oral isotretinoin therapy, particularly when assessed using dermatology- or acne-specific instruments such as DLQI, CADI, Skindex-16, and Acne-QoL. Improvements were reported across multiple domains, including emotional well-being, social functioning, and daily activities.

**Table 2:** Quality-of-life outcomes following oral isotretinoin therapy in patients with acne ^*^For Bhushan et al., the reported DLQI values (13.2 ± 3.7 at baseline and 4.2 ± 2.4 at follow-up) were extracted from the narrative results (p. 148). These values are identical to those reported by Marron et al. in a study of 346 patients from Spain. Because the source and independence of these numerical data could not be established from the published report, the Bhushan et al. values were flagged during the data audit and were not treated as independent quantitative data in the meta-analysis pending clarification QoL scores are reported as presented in the original publications; where standard deviations or sufficient numerical data were not available, the study was retained for qualitative synthesis but was not included in quantitative pooling. For DLQI, CADI, and Skindex-16, lower scores indicate better QoL, whereas higher scores indicate better QoL for SF-36 and Acne-QoL. Negative changes therefore indicate improvement for DLQI, CADI, and Skindex-16, while positive changes indicate improvement for SF-36 and Acne-QoL Acne-QoL: Acne-Specific Quality of Life Questionnaire; APSEA: Acne Patient Symptom Assessment; CADI: Cardiff Acne Disability Index; DLQI: Dermatology Life Quality Index; SD: standard deviation; SF-36: Short Form-36 Health Survey; WHOQOL-BREF: World Health Organization Quality of Life–Brief Version

Study	QoL instrument(s)	Assessment time points	Baseline QoL (mean ± SD)	Post-treatment QoL (mean ± SD)	Change in QoL	Key findings
Ng et al., 2002 [14]	WHOQOL-BREF	Baseline; 1, 3, and 6 months	Psychological: 71.9 ± 16.5; Physical: 82.2 ± 12.3; Social: 70.9 ± 19.7; Environmental: 77.4 ± 13.0	No significant change across domains	No significant change	QoL domain scores did not significantly improve despite marked clinical acne improvement; early worsening in physical QoL at 1 month
Hahm et al., 2009 [27]	APSEA (acne-related QoL)	Baseline; 2 weeks; 8 weeks	63.7 (SD not reported)	Improved at 8 weeks (exact mean not reported)	↓ Significant	Acne-related QoL improved significantly after 8 weeks; improvement correlated with reduced depressive symptoms rather than acne severity
Kaymak et al., 2009 [28]	DLQI	Baseline; 2 months; 4 months	6.11 ± 4.13	3.25 ± 3.48 (4 months)	−2.86 points	QoL improved significantly in the isotretinoin group compared with topical therapy, with greater improvement by month 4
McGrath et al., 2010 [29]	WHOQOL-BREF	Baseline; follow-up	Reduced QoL compared with controls	Significant improvement in physical and social domains	↑ Improved	Isotretinoin was associated with greater QoL improvement than antibiotic/topical therapy, particularly in social functioning
Berg and Lindberg, 2011 [30]	DLQI	Baseline; 6 months	8.55 ± 5.85	4.29 ± 4.90	−4.26 points	QoL significantly improved after treatment; isotretinoin group showed greater DLQI improvement than antibiotics or local therapy
Cyrulnik et al., 2012 [31]	Acne-QoL	Retrospective pre- vs post-treatment (telephone survey)	Self-perception domain: 9.3	Self-perception domain: 17.6	+8.3 points (domain score)	Significant improvement across Acne-QoL domains (self-perception, role-social, symptoms); role-emotional domain showed a positive trend
Ergun et al., 2012 [32]	CADI	Baseline; 1 month; 3 months; end of treatment	6.0 ± 2.7	1.7 ± 2.2	−4.3 points	Quality of life improved significantly in parallel with acne clearance; no deterioration in mood or cognitive function observed
Yesilova et al., 2012 [33]	SF-36	Baseline; 6 months	Reduced social functioning and mental health domains	Improved pain and social functioning domains	↑ Improved	Significant improvement in QoL and social anxiety scores following isotretinoin therapy
Karadag et al., 2013 [34]	SF-36	Baseline; 6 months	Social functioning: 49.1 ± 12.4	Social functioning: 56.5 ± 16.1	+7.4 points	Significant improvement in social functioning; other SF-36 domains remained unchanged
Marron et al., 2013 [35]	DLQI; SF-36	Baseline; end of treatment (30 weeks)	DLQI: 13.2 ± 3.7	DLQI: 4.2 ± 2.4	−9.0 points	DLQI values extracted from the main text, p. 704, “Quality of life, general health and psychological data.” Study population: 346 patients in Spain
Fakour et al., 2014 [36]	DLQI	Baseline; 16 weeks	7.98 ± 5.15	2.44 ± 0.60	−5.54 points	Significant improvement in QoL after isotretinoin therapy in both sexes (p = 0.001)
Rea et al., 2018 [37]	DLQI; WHOQOL-BREF	Baseline; 2 weeks	Not reported separately	Not reported separately	Not estimable	No short-term deterioration in QoL observed; study primarily demonstrated feasibility rather than treatment effect
Secrest et al., 2020 [38]	Skindex-16	Monthly (baseline to 6 months)	39.4	8.9 (month 4)	−30.5 points	Marked improvement in QoL, with >50% reduction in Skindex-16 scores by month 2; greatest improvement seen early in treatment
Bhushan et al., 2024 [39]	DLQI; CADI	Baseline; 3 months; 6 months	DLQI: 13.2 ± 3.7^*^	DLQI: 4.2 ± 2.4^*^	−9.0 points^*^	Values reported narratively on p. 148 of the original article. The numerical values are identical to those reported by Marron et al. (2013); therefore, they were flagged during data audit and not treated as independent quantitative data pending clarification. Study population: 85 patients in India

In contrast, studies using generic QoL instruments, such as WHOQOL-BREF and SF-36, demonstrated more variable findings, with some reporting modest or domain-specific improvements and others showing no statistically significant change. This distinction between generic and dermatology- or acne-specific QoL instruments highlights the importance of selecting outcome measures that are sensitive to the specific impact of acne and its treatment on patients’ quality of life. The DLQI findings were quantitatively synthesized in four studies with sufficient independent data, while the reported numerical DLQI data from Bhushan et al. (2024) were not included in the quantitative pooling because they were identical to values reported by Marron et al. (2013) and their independence could not be established.

Quantitative Synthesis

Four studies reporting DLQI outcomes with sufficient quantitative data were included in the meta-analysis (Figure 2). Pooled analysis using a random-effects model demonstrated a significant improvement in DLQI scores following oral isotretinoin treatment, with an SMD of −1.49 (95% CI: −2.48 to −0.51). Substantial heterogeneity was observed (I² = 98.1%; τ² = 0.9834; Q = 216.11, p < 0.001), reflecting differences in study design, acne severity, treatment duration, and isotretinoin dosing.

**Figure 2:**
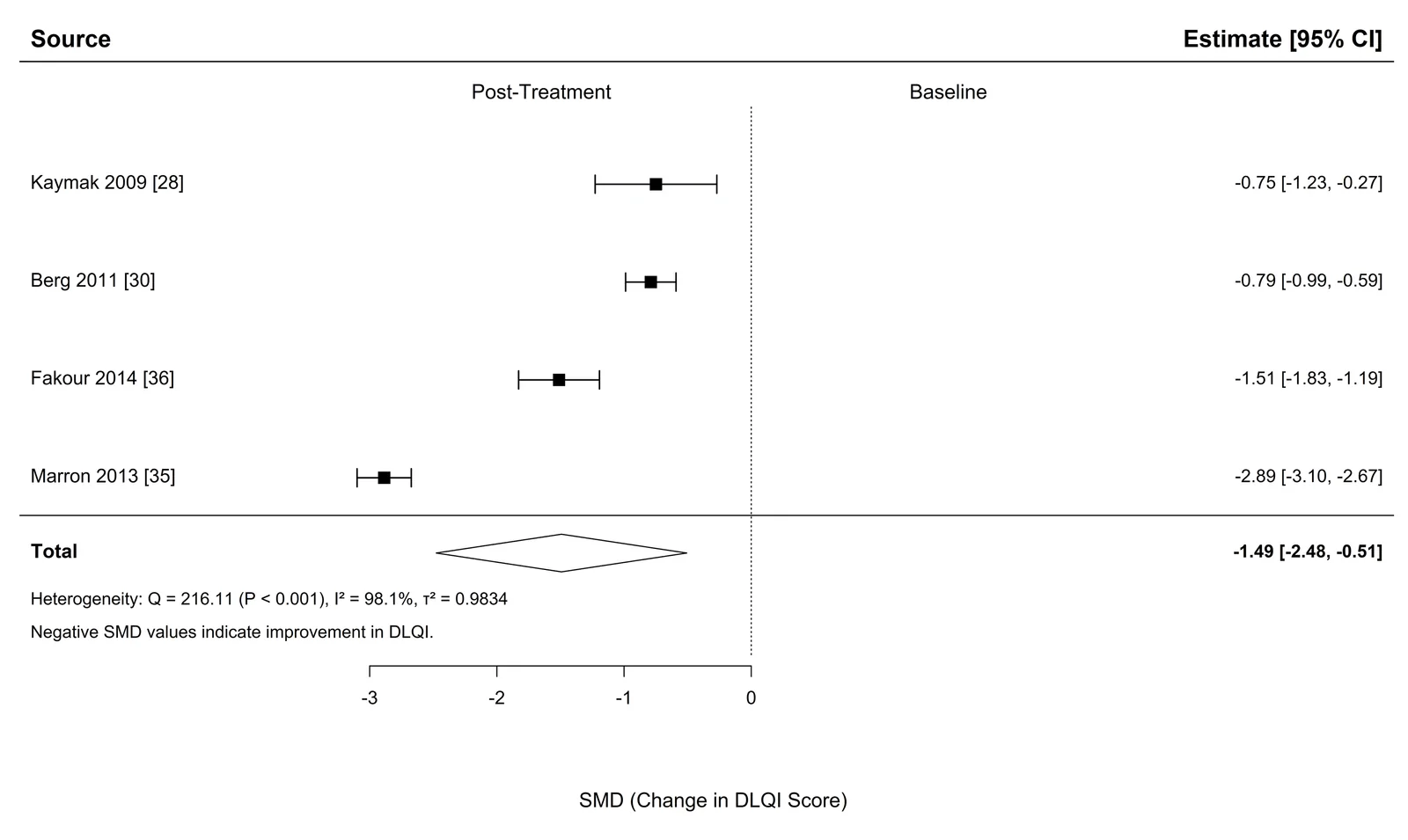
Effect of oral isotretinoin on DLQI scores in patients with acne: a random-effects meta-analysis CI: confidence interval; DLQI: Dermatology Life Quality Index; SMD: standardized mean difference

Subgroup Analysis by Acne Severity

An exploratory subgroup analysis was performed to assess whether the effect of oral isotretinoin on quality of life differed according to acne severity. Three studies including patients with mixed acne severity (mild to severe) were pooled, yielding an SMD of −1.48 (95% CI: −2.88 to −0.09), indicating a significant improvement in DLQI scores. This subgroup demonstrated substantial heterogeneity (I² = 98.8%; τ² = 1.4928; Q = 212.47, p < 0.001). Two studies restricted to patients with severe or treatment-resistant acne were also pooled and showed a comparable improvement in quality of life, with an SMD of −1.58 (95% CI: −1.84 to −1.32).

No significant heterogeneity was observed in this subgroup (I² = 0%; τ² = 0; Q = 0.58, p = 0.446). The overall test for subgroup differences was not statistically significant (Q = 0.02, p = 0.885), indicating no evidence that acne severity modified the effect of oral isotretinoin on DLQI scores. Across all five studies included in the subgroup analysis, overall heterogeneity remained substantial (SMD = −1.54, 95% CI: −2.32 to −0.76; I² = 97.1%; τ² = 0.7549; Q = 213.48, p < 0.001). Given the small number of studies within each subgroup, these findings should be interpreted with caution. The results of this exploratory analysis are presented in Figure 3.

**Figure 3:**
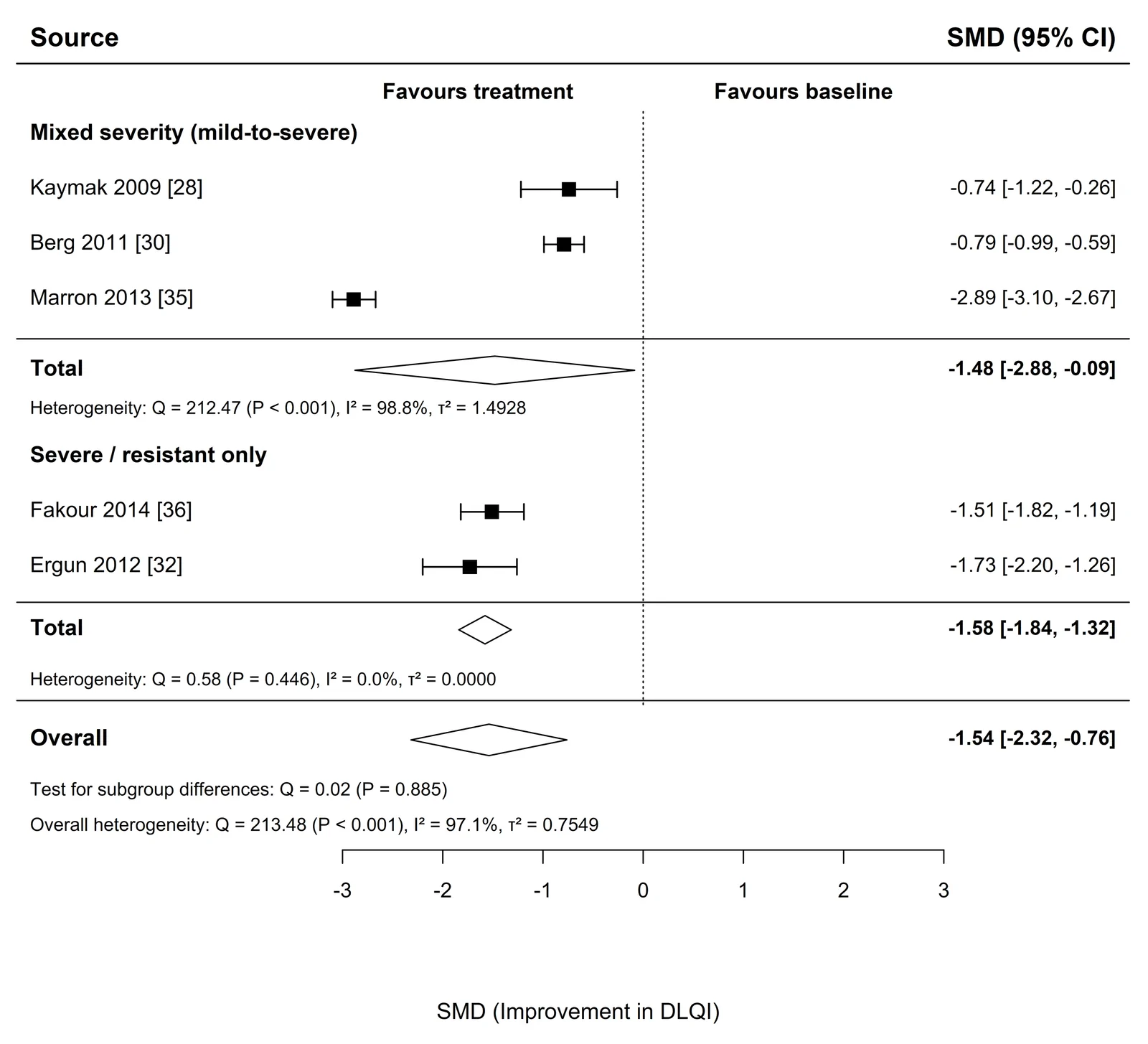
Subgroup analysis of the effect of oral isotretinoin on DLQI scores by acne severity CI: confidence interval; DLQI: Dermatology Life Quality Index; SMD: standardized mean difference

Risk-of-Bias Assessment

Risk of bias for the 13 non-randomized studies was evaluated using the ROBINS-I tool, with domain-level judgments summarized in Figure 4. Overall, most studies were judged to have moderate to serious risk of bias, primarily due to limitations inherent to observational study designs. Bias due to confounding (D1) was common across studies, reflecting the absence of randomization and limited adjustment for key clinical and psychosocial factors such as baseline acne severity, prior treatments, and mental health status. Bias in selection of participants (D2) was frequently rated as serious, particularly in retrospective and single-arm cohort studies, where inclusion depended on treatment initiation or clinic attendance.

**Figure 4:**
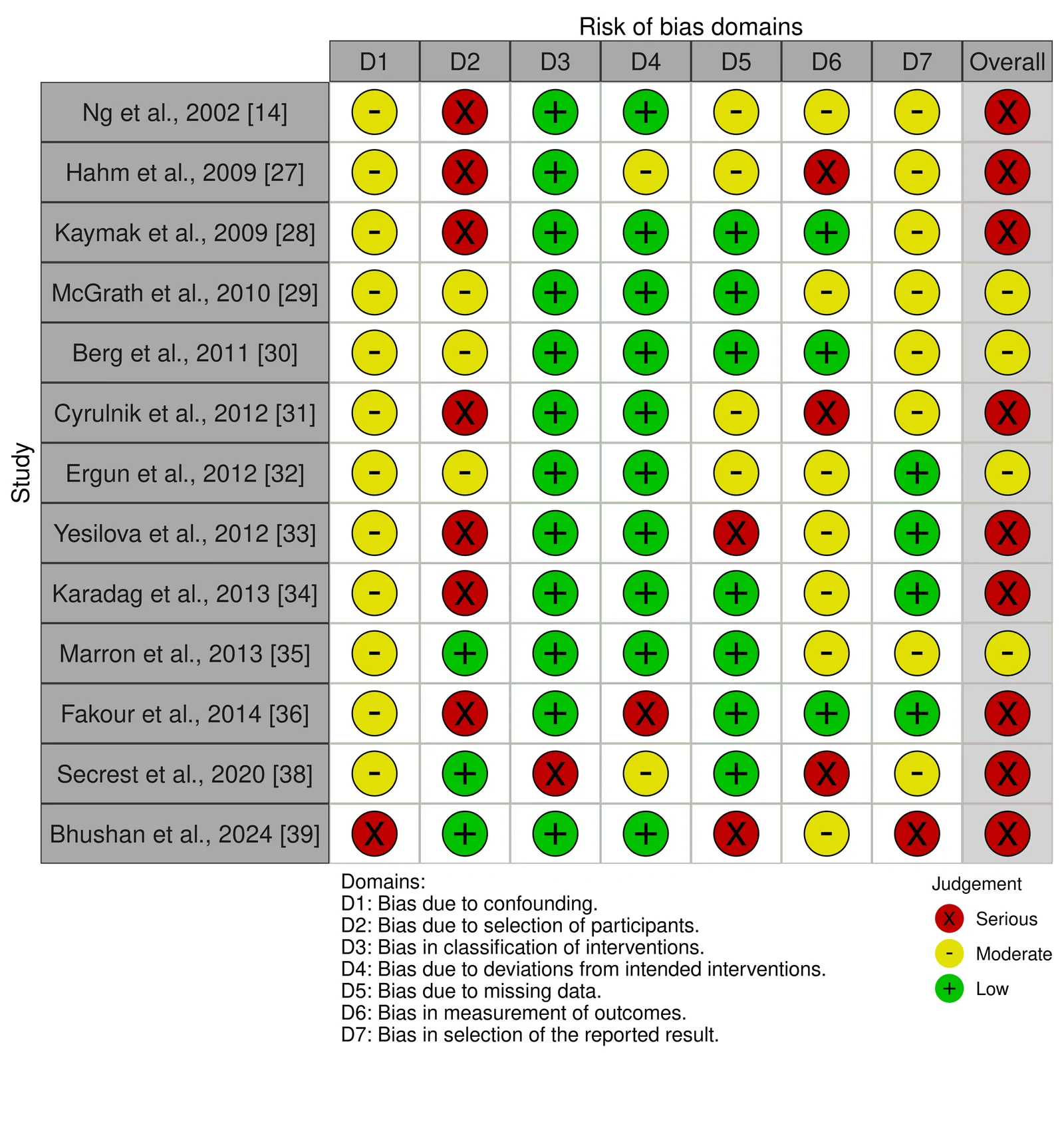
Risk-of-bias assessment of included non-randomized studies using the ROBINS-I tool ROBINS-I: Risk Of Bias In Non-randomized Studies of Interventions

Bias in classification of interventions (D3) was generally low, as oral isotretinoin exposure was clearly defined. Bias due to deviations from intended interventions (D4) was typically low to moderate, with most studies adhering to standard dosing regimens. Missing data (D5) and measurement of outcomes (D6) contributed additional concerns, as quality-of-life outcomes were self-reported and follow-up completeness varied. Selective reporting bias (D7) was assessed as moderate in several studies due to the absence of preregistered protocols. The single randomized controlled trial was assessed separately using the Cochrane RoB 2 tool and was judged to have some concerns overall [37].

Discussion

This systematic review and meta-analysis evaluated the effect of oral isotretinoin therapy on quality of life in patients with acne, integrating evidence from fourteen studies published over more than two decades. The findings demonstrate that oral isotretinoin is associated with a clinically meaningful improvement in quality of life, particularly when assessed using dermatology- and acne-specific instruments. The quantitative synthesis showed a large pooled effect on DLQI scores, with an SMD of −1.49 (95% CI: −2.48 to −0.51), indicating a substantial reduction in disease-related quality-of-life impairment following treatment. However, substantial heterogeneity was observed (I² = 98.1%), suggesting that the magnitude of improvement varied considerably across studies, potentially reflecting differences in study design, acne severity, treatment duration, and isotretinoin dosing.

The large pooled standardized effect indicates a substantial improvement in disease-related quality of life following oral isotretinoin treatment, indicating that the benefits of oral isotretinoin extend beyond physical lesion clearance to broader psychosocial domains [40]. Acne-related disfigurement is known to contribute to emotional distress, social avoidance, and depressive symptoms, particularly among adolescents and young adults during sensitive developmental periods [41]. Effective treatment with isotretinoin may therefore alleviate these burdens by improving appearance, self-confidence, and social functioning, as reflected by consistent improvements across acne-specific quality-of-life instruments such as DLQI, CADI, Skindex-16, and Acne-QoL [31].

At the same time, concerns regarding potential isotretinoin-associated mood changes remain clinically relevant. Epidemiological studies have reported variable rates of depressive symptoms during treatment, ranging from approximately 1-11%, although causality has not been clearly established [42]. Proposed biological mechanisms include central nervous system penetration and modulation of neurotransmitter systems involved in mood regulation. However, conflicting evidence suggests that improvement in acne severity itself may have a protective or therapeutic effect on acne-associated psychological distress [42]. Together, these findings underscore the importance of interpreting quality-of-life improvements alongside careful mental health monitoring during isotretinoin therapy.

In contrast, studies using generic quality-of-life instruments such as WHOQOL-BREF and SF-36 demonstrated more variable results. Some reported modest or domain-specific improvements, while others found no statistically significant change. This discrepancy likely reflects the lower sensitivity of generic instruments to dermatology-specific concerns [43]. Acne-related distress is often concentrated in appearance-related, social, and emotional domains, which may not be adequately captured by broad health-related quality-of-life measures [44]. These findings support the preferential use of dermatology- or acne-specific instruments when evaluating patient-reported outcomes in acne research.

Substantial heterogeneity was observed in the primary meta-analysis, with an I² value exceeding 90%. This degree of heterogeneity is not unexpected given the diversity of included study designs, patient populations, isotretinoin dosing regimens, and follow-up durations. Differences in baseline acne severity, prior treatment exposure, and psychosocial vulnerability may further contribute to variability in quality-of-life responses. Despite this heterogeneity, the direction of effect was consistently favorable across all studies included in the quantitative synthesis, strengthening confidence in the overall finding of benefit.

The exploratory subgroup analysis by acne severity did not identify a statistically significant difference between studies enrolling patients with mixed severity acne and those restricted to severe or treatment-resistant disease. Both subgroups demonstrated large improvements in DLQI scores, with pooled effect estimates of similar magnitude. Notably, heterogeneity was minimal among studies focusing exclusively on severe acne, whereas it remained high in mixed-severity cohorts. This may reflect greater clinical homogeneity and more pronounced baseline impairment among patients with severe disease [42]. However, the limited number of studies within each subgroup necessitates cautious interpretation, and these results should be viewed as hypothesis-generating rather than confirmatory.

The findings of this review contribute to the ongoing debate regarding the net impact of isotretinoin on patient well-being. While concerns regarding psychiatric adverse effects have been raised in the literature, the overall pattern of evidence synthesized here suggests that improvements in acne severity are accompanied by improvements in quality of life for most patients. This observation is consistent with the concept that effective disease control may alleviate psychological distress related to appearance, social stigma, and self-esteem. Importantly, none of the included studies reported a sustained deterioration in quality of life attributable to isotretinoin treatment.

Risk-of-bias assessment revealed that most non-randomized studies were subject to moderate or serious risk of bias, particularly related to confounding and participant selection. These limitations reflect the inherent challenges of observational research in dermatology, where randomized trials may be difficult to conduct for ethical or practical reasons. Nonetheless, intervention classification was generally robust, and outcome measures were based on validated instruments, lending credibility to the observed associations.

The findings of this review indicate that oral isotretinoin provides effective treatment for moderate to severe acne while also producing substantial improvements in patient-reported quality of life. These benefits appear to extend beyond clinical clearance to psychosocial domains, including emotional well-being and social functioning, which are particularly relevant in adolescents and young adults. Clinicians should therefore incorporate quality-of-life outcomes into therapeutic decision-making, especially for patients experiencing significant psychological or social distress related to acne. The observed benefits across different acne severity levels support the use of isotretinoin when clinically indicated, provided that patients are carefully selected and appropriately monitored. Routine use of validated dermatology-specific quality-of-life instruments may assist in identifying patients most likely to benefit and in facilitating shared decision-making. At the same time, thorough counseling regarding potential adverse effects and ongoing assessment of mental well-being remain essential to ensure safe and patient-centered care.

Limitations

This study has several limitations that should be considered when interpreting the findings. First, the majority of included studies were observational in design, which increases susceptibility to confounding and selection bias despite the use of validated risk-of-bias assessment tools. Second, substantial heterogeneity was observed in the quantitative synthesis, likely reflecting differences in study design, acne severity, isotretinoin dosing regimens, and follow-up duration. Third, only four studies provided sufficient data for inclusion in the meta-analysis, limiting statistical power and precluding formal assessment of publication bias. Fourth, quality-of-life outcomes were assessed using a variety of instruments, which may reduce comparability across studies, particularly between acne-specific and generic measures. Finally, most studies relied on self-reported outcomes, which are subject to reporting bias and may be influenced by patient expectations. These limitations highlight the need for cautious interpretation of the pooled estimates.

## Conclusions

This systematic review and meta-analysis demonstrates that oral isotretinoin therapy is associated with significant and clinically meaningful improvements in quality of life among patients with acne, particularly when assessed using dermatology- and acne-specific instruments. The pooled analysis of DLQI outcomes showed a large reduction in quality-of-life impairment following treatment. However, the substantial heterogeneity across studies and the predominance of non-randomized study designs warrant cautious interpretation of the pooled effect. Exploratory subgroup analyses found no statistically significant difference in treatment effect according to acne severity, although the small number of studies within each subgroup limits the strength of this finding. While potential adverse effects, including psychiatric symptoms, warrant appropriate clinical monitoring, the available evidence supports oral isotretinoin as a treatment associated with improved patient-reported quality of life in patients with acne. Further high-quality prospective and randomized studies using standardized quality-of-life instruments, clearly defined acne-severity categories, and longer follow-up are needed to confirm the magnitude and durability of these benefits and to identify patients most likely to derive the greatest quality-of-life improvement.

## Appendices

Appendix 1. Complete electronic database search strategies

The following search strategies were used to identify potentially eligible studies. The searches were conducted from database inception to April 2026.

PubMed (MEDLINE)

(acne[Title/Abstract] OR "acne vulgaris"[Title/Abstract] OR "Acne Vulgaris"[Mesh]) AND (isotretinoin[Title/Abstract] OR "oral isotretinoin"[Title/Abstract] OR Accutane[Title/Abstract] OR Roaccutane[Title/Abstract] OR "13-cis-retinoic acid"[Title/Abstract] OR "Isotretinoin"[Mesh]) AND ("quality of life"[Title/Abstract] OR QoL[Title/Abstract] OR HRQoL[Title/Abstract] OR "health-related quality of life"[Title/Abstract] OR DLQI[Title/Abstract] OR "Dermatology Life Quality Index"[Title/Abstract] OR CADI[Title/Abstract] OR "Cardiff Acne Disability Index"[Title/Abstract] OR "Acne-QoL"[Title/Abstract] OR Skindex[Title/Abstract] OR "WHOQOL-BREF [Title/Abstract] OR "SF-36"[Title/Abstract])

Scopus

TITLE-ABS-KEY (acne OR "acne vulgaris") AND TITLE-ABS-KEY (isotretinoin OR "oral isotretinoin" OR accutane OR roaccutane OR "13-cis-retinoic acid") AND TITLE-ABS-KEY ("quality of life" OR QoL OR HRQoL OR "health-related quality of life" OR DLQI OR "Dermatology Life Quality Index" OR CADI OR "Cardiff Acne Disability Index" OR "Acne-QoL" OR Skindex OR "WHOQOL-BREF" OR "SF-36")

Web of Science Core Collection

TS=(acne OR "acne vulgaris") AND TS=(isotretinoin OR "oral isotretinoin" OR accutane OR roaccutane OR "13-cis-retinoic acid") AND TS=("quality of life" OR QoL OR HRQoL OR "health-related quality of life" OR DLQI OR "Dermatology Life Quality Index" OR CADI OR "Cardiff Acne Disability Index" OR "Acne-QoL" OR Skindex OR "WHOQOL-BREF" OR "SF-36")

Cochrane Library

("acne vulgaris") AND (isotretinoin) AND ("quality of life" OR QoL OR HRQoL OR DLQI OR CADI OR "Acne-QoL" OR Skindex OR "WHOQOL-BREF" OR "SF-36")

Google Scholar

Google Scholar was searched using combinations of terms related to acne, oral isotretinoin, and quality of life, including:

"acne vulgaris" AND isotretinoin AND "quality of life"

Additional combinations of relevant terms, including "acne", "oral isotretinoin", "DLQI", "CADI", "Acne-QoL", "Skindex", "WHOQOL-BREF", and "SF-36", were used to identify potentially relevant studies. Reference lists of included studies were also screened manually.
